# Supplementary figures and images for: Building the cytokinetic contractile ring in an early embryo: Initiation as clusters of myosin II, anillin and septin, and visualization of a septin filament network
Source: PLoS One. 2021 Dec 28;16(12):e0252845. doi: 10.1371/journal.pone.0252845 (PMC8714119; doi:10.1371/journal.pone.0252845)

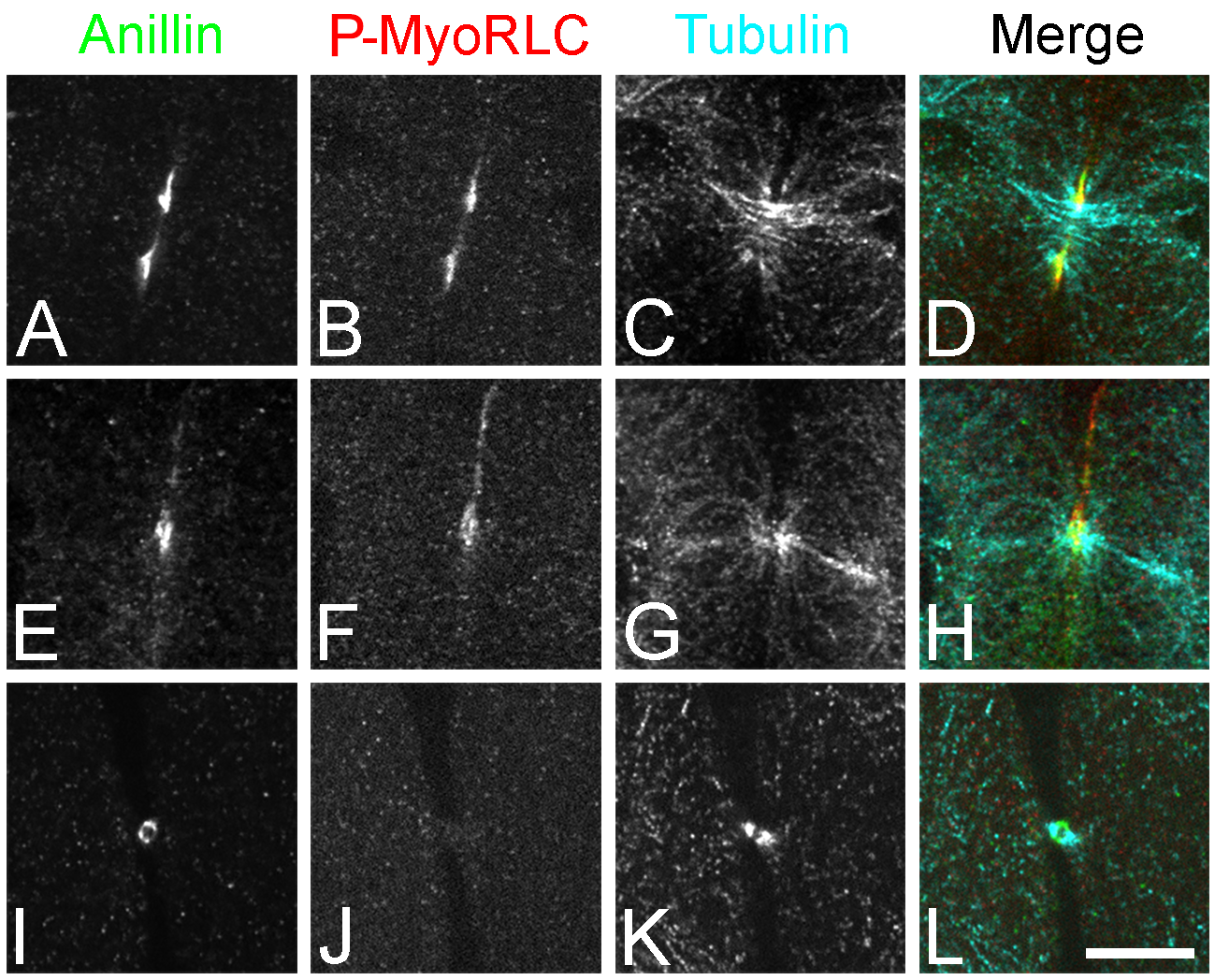

Supplement: S1 Fig — Anillin (A, E, and I—green), P-MyoRLC (B, F, and J—red), and microtubules (C, G, and K—cyan) were imaged in S. purpuratus embryos at the end of cytokinesis by confocal microscopy. In the late ingressing embryos (A-H), anillin and P-MyoRLC are enriched in the contractile ring (A-D), as well as the forming midbody (E-H). Anillin remains associated with the midbody after the contractile ring has completed constriction and P-MyoRLC staining is lost (I-L). Bar, 15 μm. (TIF) [file pone.0252845.s001.tif]

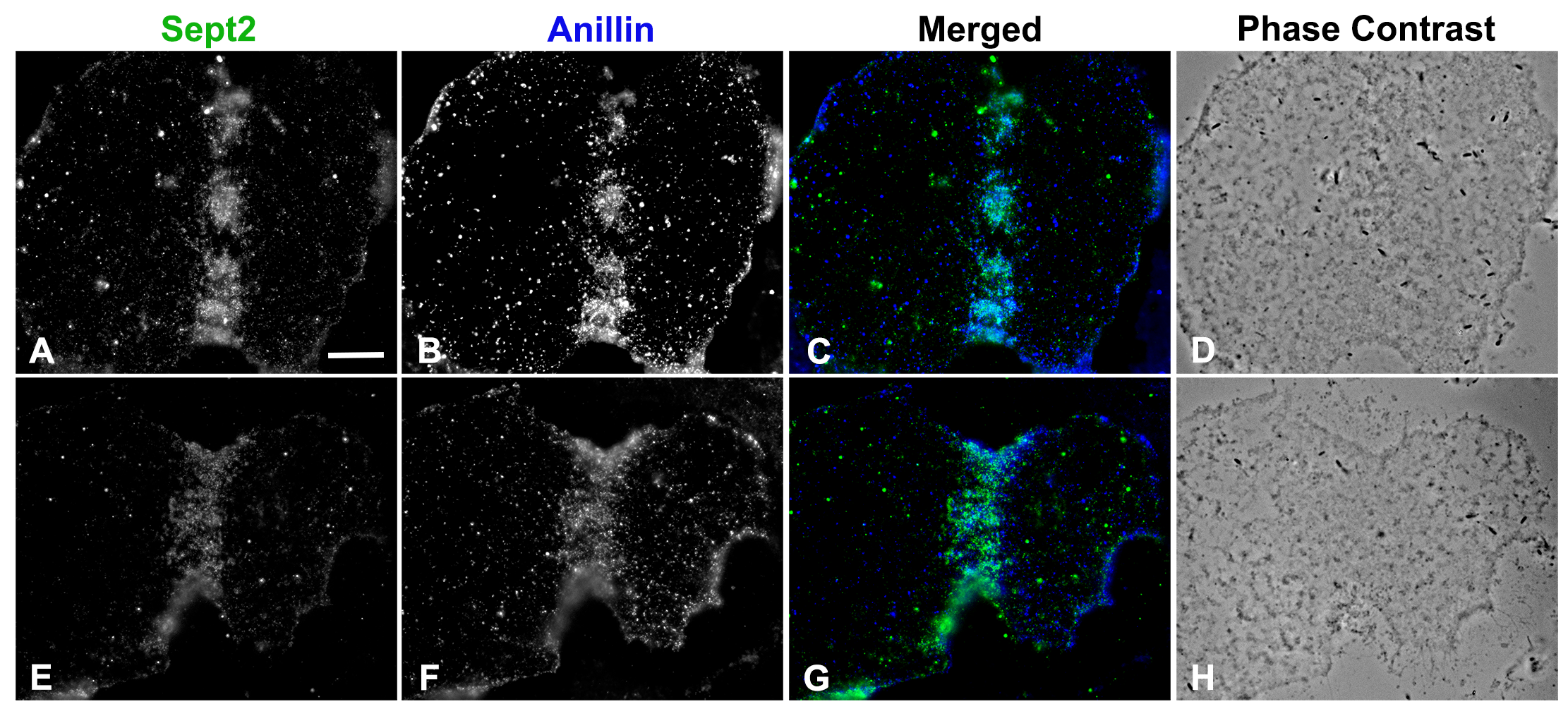

Supplement: S2 Fig — Sept2 (A, E, C, and G—green) and anillin (B, F, C, and G—blue) antibodies labeled with the Zenon rabbit IgG labeling kit (Molecular Probes) show a general colocalization within the CR region of double labeled mid-late stage isolated cortices from S. purpuratus. Bar in A = 10 μm, magnifications of A-H are equivalent. (TIF) [file pone.0252845.s002.tif]

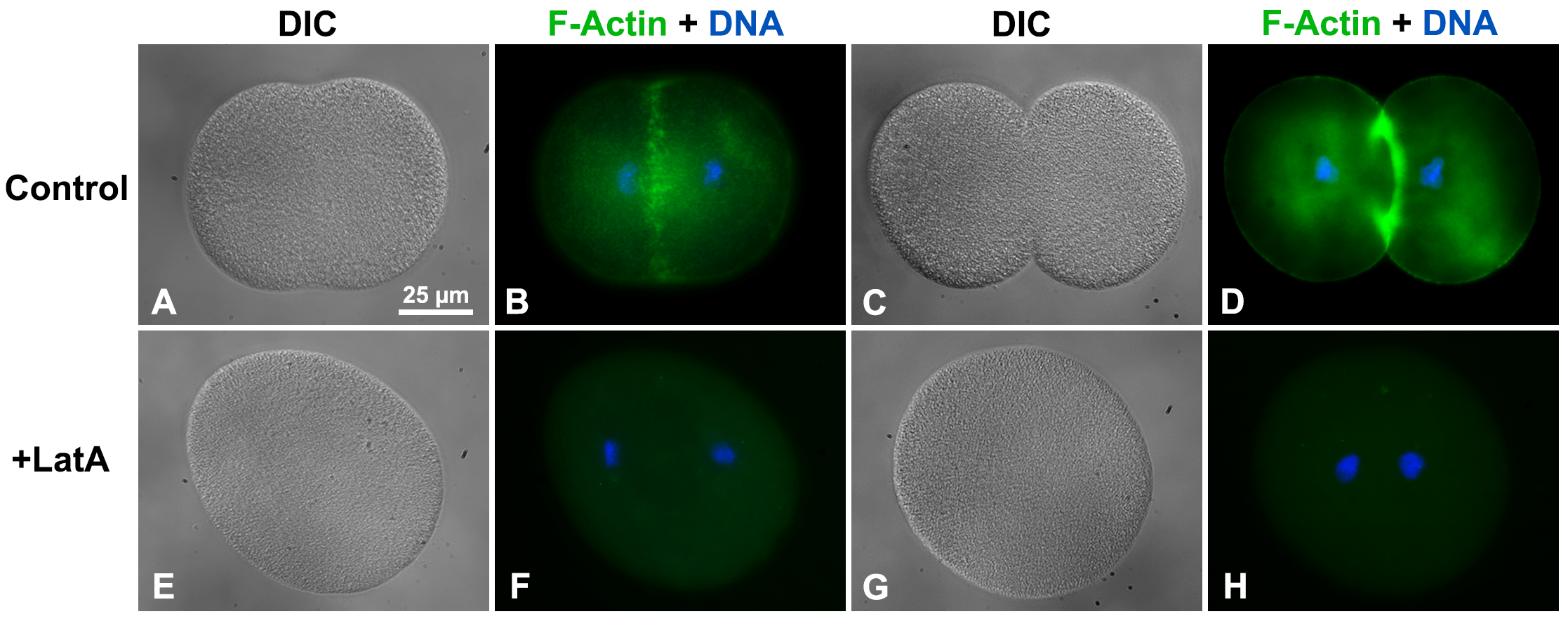

Supplement: S3 Fig — Staining of control (A-D) and LatA treated (E-H) whole S. purpuratus embryos with fluorescent phalloidin (green) and DAPI (blue) at equivalent time points shows LatA-mediated loss of actin filaments and inhibition of cytokinesis–but not karyokinesis. The control embryo in panel B shows F-actin in the clusters stage of CR organization, whereas the later stage control embryo in panel D shows a clear linearized ring. The cortical microvilli-associated phalloidin staining present in control embryos is not seen in the LatA treated embryos. Bar in A = 10 μm, magnifications of A-H are equivalent, and all images are widefield. (TIF) [file pone.0252845.s003.tif]
