# Supplementary material for: Building the cytokinetic contractile ring in an early embryo: Initiation as clusters of myosin II, anillin and septin, and visualization of a septin filament network
Source: PLoS One. 2021 Dec 28;16(12):e0252845. doi: 10.1371/journal.pone.0252845 (PMC8714119; doi:10.1371/journal.pone.0252845)
Supplement: S1 File — Panel A is the full anti-anillin immunoblot from Fig 1B. Lane 1 = PH domain immunogen; Lane 2 = S. purpuratus egg; Lane 3 = S. purpuratus first division embryo. Panel B corresponds to the total protein Poncea S stained anti-Sept2 blot from Fig 1C. Panel C corresponds to Fig 1C anti-Sept2 immunoblot of the original blot destained for total protein. In panels B and C: Lane 1 = L. pictus first division embryo; Lane 2 = LLC-PK1 cells; Lane 3 = Prestained molecular weight standards. (PDF) [file pone.0252845.s004.pdf]

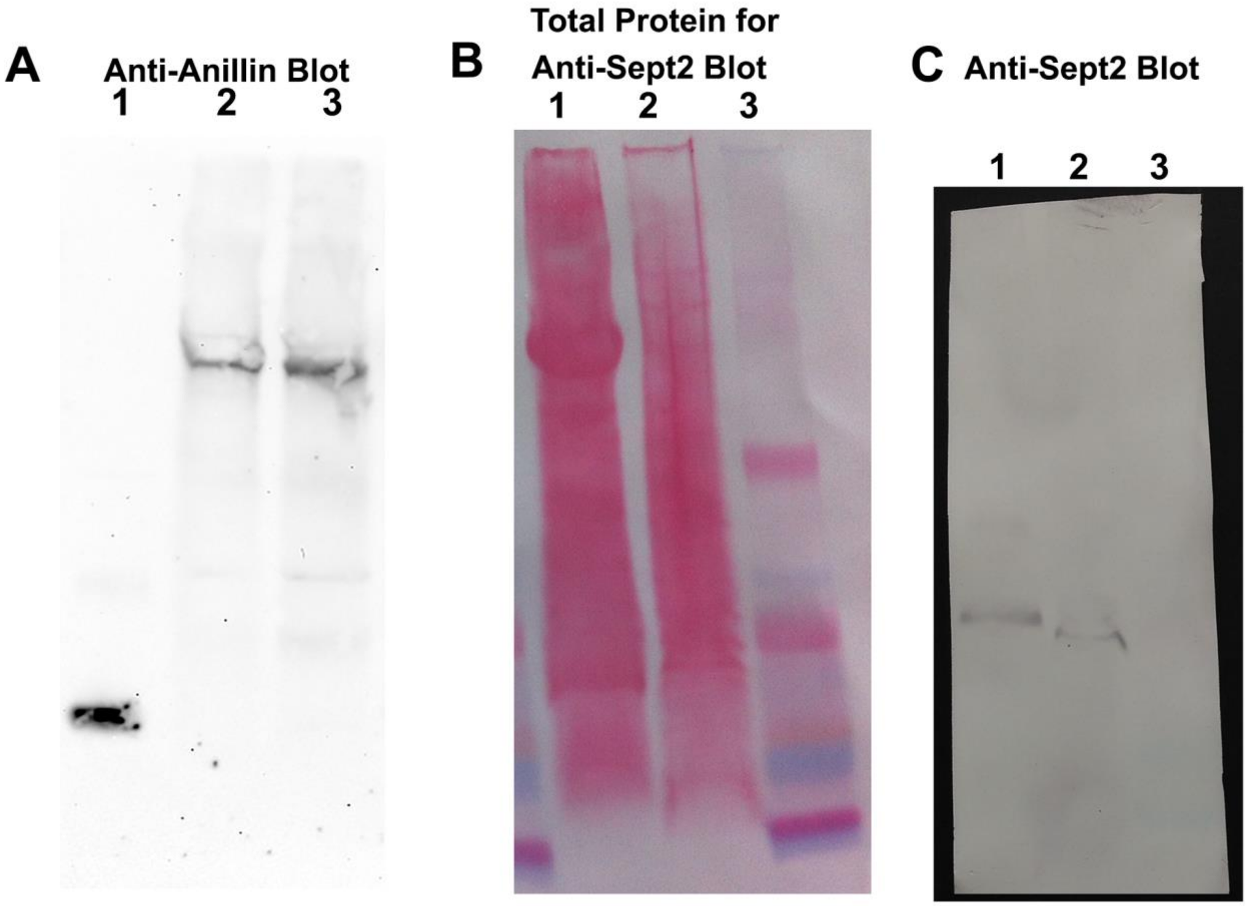

S1 File. Original immunoblots from Figs 1B and 1C. Panel A is the full anti-anillin immunoblot from Fig 1B. Lane 1 = PH domain immunogen; Lane 2 = *S. purpuratus* egg; Lane 3 = *S. purpuratus* first division embryo. Panel B corresponds to the total protein Poncea S stained anti-Sept2 blot from Fig 1C. Panel C corresponds to Fig 1C anti-Sept2 immunoblot of the original blot destained for total protein. In panels B and C: Lane 1 = *L. pictus* first division embryo; Lane 2 = LLC-PK1 cells; Lane 3 = Prestained molecular weight standards.
